# Analysis of the effects of Zhenju antihypertensive tablet on efficacy, safety and vascular endothelial function in patients with essential hypertension: A protocol for systematic review and meta-analysis

**DOI:** 10.1097/MD.0000000000027501

**Published:** 2021-11-19

**Authors:** Jisen Zhao, Dong Guo, Maoxia Fan, Yongcheng Liu

**Affiliations:** a The First Clinical Medical College of Shandong University of Traditional Chinese Medicine, Jinan City, Shandong Province, China; b Teacher Development Center of Shandong University of Traditional Chinese Medicine, Jinan City, Shandong Province, China; c Shandong Provincial Hospital of Traditional Chinese Medicine, Jinan, Shandong Province, China.

**Keywords:** endothelial function, essential hypertension, meta-analysis, regimen, systematic review, Zhenju antihypertensive tablet

## Abstract

**Background:**

: As a compound preparation of traditional Chinese and western medicine included in Volume 20 of Chinese traditional Medicine prescription, Zhenju antihypertensive tablet has been widely used in the treatment of patients with essential hypertension (EH) for many years. This study intends to evaluate the efficacy, safety and vascular endothelial function of Zhenju antihypertensive tablet in the treatment of essential hypertension.

**Methods:**

: The search strategies of different websites were searched on Cochrane Central controlled Trials Registry, PubMed, excerpt database, Chinese Biomedical Literature Database, China National knowledge Infrastructure, Chinese Science and Technology Journal Database, WanFang, and other websites. All qualified studies were confirmed to include randomized controlled trials. The search time range was from January 1, 1900 to August 31, 2021. At the same time, the list of references and related reviews were checked. Two evaluators were responsible for the extraction and management of the data independently. The literature quality was evaluated according to Cochrane manual 4.2.2. Heterogeneity test and Meta analysis were carried out by Review ManagerV.5.3 software. The bias risk included in the study was evaluated by Cochrane “bias risk” tool. In addition, the relevant statistical data were evaluated by GRADE3.6 evidence quality grading system.

**Results:**

: This study intends evaluate the efficacy and safety of Zhenju antihypertensive tablet in the treatment of EH from 4 aspects, including changes in blood pressure (systolic blood pressure, diastolic Blood pressure), effective hypotension, changes in endothelial function (NO, the level of plasma endothelin-1 in serum), and adverse reactions.

**Conclusion:**

: The conclusion of this study intends to provide evidence for judging the effectiveness and safety of ZJAHC intervention on EH patients and their endothelial function.

PROSPERO registration number: PROSPERO CRD42021235309.

## 1 Introduction

As the main cause of death and disability in the world, cardiovascular disease in China accounts for 45.91% and 43.56% of the causes of death in rural and urban areas respectively, and mortality occupies the first place. Hypertension is the largest and most interferable risk factor for cardiovascular disease.^[1]^ Long-term hypertension can cause major target organ damage, lead to stroke, myocardial infarction, chronic kidney disease and other serious complications with high disability rate and mortality, which brings heavy medical and economic burden to individual families and countries.^[2]^ At present, hypertension patients who are unable to confirm the specific causes of elevated blood pressure are defined as essential hypertension (EH) patients with essential hypertension, accounting for 900.95% of the total number of hypertension patients (about 245 million).^[3]^ According to the data released by the 2017 guidelines for rational Drug use of Hypertension (2nd Edition), the prevalence rate of hypertension in adults over 18 years old in China is as high as 25.2% (about 244500). However, the blood pressure standard rate of hypertensive patients after treatment is only 29.6%. The disease control rate and treatment standard rate are still at a lower level compared with the United States.^[4,5]^ The comprehensive prevention and treatment of hypertension has become one of the main public health problems in our country at present. Vascular endothelium is the target organ of a variety of cardiovascular diseases or risk factors. Endothelial dysfunction is often associated with and aggravating cardiovascular disease. Vascular endothelial dysfunction is related to atherosclerosis, hypertension and other diseases and states. Studies have shown that the evaluation of endothelial function can make early, accurate and comprehensive diagnosis of cardiovascular diseases.^[6,7]^ The changes of vascular endothelial function are often reflected by the detection of the content of endothelial secretion nitric oxide level in serum and the level of plasma endothelin-1 in serum in serum in clinic.^[8]^

Zhenju antihypertensive tablet was independently developed by China and has been widely used in clinic since 1960 s. So far, it has accumulated a large number of clinical data.^[9]^ Zhenju antihypertensive tablet is a compound preparation of traditional Chinese and western medicine composed of wild chrysanthemum, pearl layer powder, rutin, clonidine hydrochloride (CLO) and hydrochlorothiazide (HCT).^[10]^ The pearl layer powder in Zhenju antihypertensive tablet and wild chrysanthemum work together to calm the liver, clear heat, and reduce fire.^[11]^ Clonidine hydrochloride can reduce blood pressure by reducing sympathetic activity, peripheral vascular resistance and heart rate. Hydrochlorothiazide is a thiazide diuretic. In the early stage, hydrochlorothiazide can reduce blood pressure by reducing blood volume through natriuretic diuresis, and hydrochlorothiazide can also eliminate sodium retention caused by clonidine hydrochloride. Rutin can reduce capillary permeability, increase its tension, and reduce the level of serum triglyceride.^[12]^ It is worth mentioning that studies have shown that the recommended dose range of HCT in conventional antihypertensive drugs is 12.5 to 50 mg, and there are obvious side effects in the course of treatment. However, the dosage of HCT used in ZJAHC is 5 mg, but its curative effect is similar to or even better than some first-line treatment drugs. It indicates that the drug composition of Zhenju antihypertensive tablet has a benign pharmacokinetic effect. It can contribute to improve the antihypertensive activity of HCT and reduce side effects.^[13]^

Zhenju antihypertensive tablet combined with conventional antihypertensive drugs in the treatment of EH has been used in clinic for many years. In addition, a large number of clinical data have been accumulated. However, there is a lack of systematic evaluation due to the single-center, small-sample randomized controlled trials. As a consequence, this scheme collates a large number of literatures for systematic evaluation and meta-analysis to evaluate the efficacy, safety and vascular endothelial function of Zhenju antihypertensive tablet in the treatment of EH patients, providing more comprehensive evidence for the clinical application of Zhenju antihypertensive tablet.

## 2 Methods

### 2.1 Inclusion criteria

#### 2.1.1 Design type

Clinical randomized controlled trial;

#### 2.1.2 Objects

The diagnostic criteria of patients with essential hypertension were in accordance with the diagnostic criteria of WHO/ISH hypertension guidelines or the 2018 revised China guidelines on Prevention and treatment of Hypertension.

#### 2.1.3 Intervention measures

The experimental group was treated with Zhenju antihypertensive tablet + routine antihypertensive drugs, and the control group was treated with conventional antihypertensive drugs or conventional antihypertensive drugs + placebo.

#### 2.1.4 Observation index

Efficacy indicators: including changes in blood pressure (systolic blood pressure, diastolic Blood pressure), antihypertensive effective rate (including effective, effective, ineffective, etc); indicators affecting vascular endothelial function: including changes in the content of nitric oxide level in serum and the level of plasma endothelin-1 in serum in plasma; safety indicators: including the incidence of adverse reactions.

### 2.2 Exclusion criteria

All uncontrolled trials and non-randomized controlled trials; historical controls (comparison of the results of studies conducted in 2 different periods); Comparison between disease and nondisease groups; Trials distributed according to patient characteristics (sex, age, disease severity, different etiology, regional distribution, etc); The control group was treated with other clinical trials of nonwestern medicine, such as other types of traditional Chinese medicine or proprietary Chinese medicine; Clinical trials with irregular evaluation indexes or without detailed publication of treatment results, clinical trials without statistical basic data; Reviews, animal experiments, special reports of adverse reactions and nonclinical efficacy studies such as pharmacology and pharmacokinetics; Obvious errors or defects in trial design.

### 2.3 Data source and retrieval

The research data came from the clinical research literature on Zhenju antihypertensive tablet in the treatment of essential hypertension published in biomedical journals from 1900 to 2021. PubMed, CochraneLibrary, excerpt database, Chinese Biomedical Literature Database, China National knowledge Infrastructure, Chinese Science and Technology Journal Database, WanFang were selected for retrieval. The WHO International Clinical Laboratory trial Registration platform (apps.who.int/trialsearch/) and Clinical Trials (www.clinicaltrials.gov) would saso be selected for ongoing studies. References that meet the inclusion criteria would be reviewed one by one to avoid omissions.

The Chinese database was searched by the combination of title or key words and subject words. In addition, the key words were as follows: the key words in English database were as follows: The search was carried out by the way of subject words combined with free words, and the specific retrieval was as follows (Table 1).

**Table 1:** Search strategy for PubMed.

Number	Search terms
1	#1 Search (“Essential Hypertension”[Mesh]) OR (“Hypertension”[Mesh]) OR (“Blood Pressure, High” [Mesh]) OR (“Blood Pressures, High”[Mesh]) OR (“High Blood Pressure”[Mesh]) OR (”High Blood Pressures" [Mesh])
2	#2 Search (“Zhenju-Jiangya Tablet”) OR (“Zhenju Jiangya Tablet”)
3	#3Search(“randomized controlled trial” [Title/Abstract]) OR (“randomized”[Title/Abstract]) OR (“randomly”[Title/Abstract])
4	#4 Search (“Endothelium, Vascular”) OR (“Endothelium”)
5	#1 AND #2 AND #3 AND#4

### 2.4 Data collection and analysis

#### 2.4.1 Literature screening

**Figure 1:**
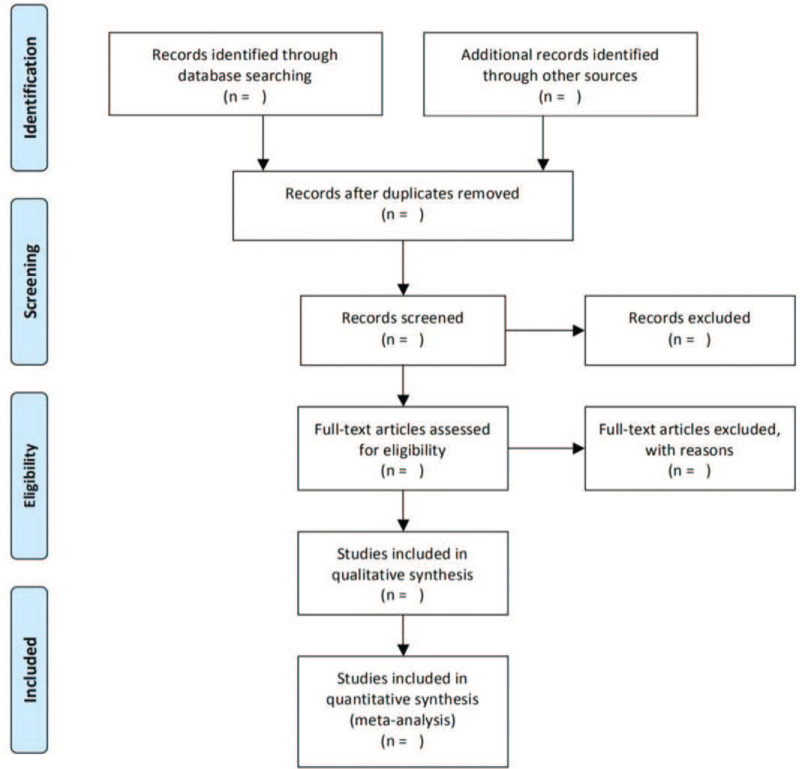
PRISMA flowchart.

The 2 researchers independently screened the literature, extracted data and quality evaluation and cross-checked them according to the established screening criteria. In case of differences, the 2 sides would resolve them through discussion or consultation with the third research member. First of all, the 2 sides read the title and abstract of the obtained literature for preliminary screening, exclude the literature that obviously does not meet the inclusion criteria, and further read the full text for re-screening to determine whether it is included or not. The literature screening process was given in the flowchart of “preferred report items for systematic review and meta-analysis” (Fig. 1).

#### 2.4.2 Extraction result

The researchers used Excel to establish a data extraction table, which included: Basic information of the literature: title, author, journal published, year; Basic situation of study subjects, grouping, course of disease, baseline, diagnosis and inclusion / exclusion criteria, randomized and blind use; Intervention measures, course of treatment; Elements of risk bias assessment; Outcome indicators: including effectiveness, safety, etc.

The author would contact the author by email if complete information was not available. If the complete data were still not available, the literature would be deleted.

#### 2.4.3 Methodological quality evaluation

The bias risk assessment tool recommended by the Cochrane system evaluator manual 5.3.0 and the improved Jadad rating scale were used to evaluate the quality of the study. In addition, the bias risk assessment tool recommended by the Cochrane system evaluator manual 5.3.0 was used to evaluate the study quality according to 7 aspects: random method, assignment hiding, subject blind method, blind result evaluation method, data integrity, selective report, and other bias. The modified Jadad score scale was used to evaluate the quality of the literature. The total score of Jadad score was 7, ≤3 for low quality literature and ≥4 for high quality literature.

#### 2.4.4 Data analysis

The collected information was statistically analyzed by Rev Man5.3.0 software. The counting data were analyzed by relative risk or ratio. In the analysis of measurement data, mean difference analysis obtained by the same measurement unit if the measured value of the outcome was based on the mean difference. In addition, if the same outcome was evaluated but measured according to different methods, the standardized mean difference analysis was used. Besides, the composite results were expressed by the effect value and its 95% confidence interval.

#### 2.4.5 Heterogeneity evaluation

The clinical heterogeneity and statistical heterogeneity between studies were judged. Clinical heterogeneity was judged according to the similarity of research objects, intervention measures, control and outcome indicators between studies. In addition, statistical heterogeneity was evaluated by *I*^2^: If *I*^2^ ≤ 50% and *P* > .1, statistical homogeneity was considered to be great and fixed effect model was used to merge. If *I*^2^ > 50% or *P* ≤ .1, the statistical heterogeneity was large, then the source of heterogeneity was further analyzed. Random effect model was used for Meta analysis after the obvious clinical heterogeneity is excluded. When there was obvious clinical heterogeneity, it should be treated by subgroup analysis or sensitivity analysis, or only descriptive analysis.

#### 2.4.6 Publication bias

The funnel chart and Eiger test should be used to analyze whether there was publication bias if a certain outcome index was included in more than 10 articles.

#### 2.4.7 Subgroup analysis

If there were at least 10 trials included, a subgroup analysis would be conducted based on different interventions, participants, gender, treatment duration and drug dose to explore the source of heterogeneity.

#### 2.4.8 Sensitivity analysis

Sensitivity analysis based on sample size, missing data and methodological quality would be conducted through “leave-one-out” to determine whether the results were affected by the use of different analysis methods (random effect model or fixed effect model) so as to support the robustness of the results.

#### 2.4.9 GRADE evidence quality classification^[14]^

The evidence quality evaluation system GRADE was used to evaluate the evidence quality, the evidence quality was divided into high quality, medium quality, low quality, very low quality for the results of this system evaluation. In addition, the recommended grade was divided into strong and weak levels.

## 3 Conclusion

In fact, it was a study to evaluate the efficacy, safety and effect of Zhenju antihypertensive tablet (ZJAHT) on vascular endothelial function in patients with EH. The first-line antihypertensive drugs which are widely used in clinic frequently appear side effects such as dizziness and nausea in the long-term use of EH patients at present. In addition, some of them are combined with dosage because of drug resistance.^[15]^ The combination of traditional Chinese and western medicine has complementary advantages, which provides more choices for effectively controlling EH, strengthening the curative effect and reducing side effects. In the meanwhile, this study puts forward the effect of ZJAHT on vascular endothelial function in patients with EH for the first time. It makes a new attempt and exploration for ZJAHT in the treatment of other serious cardiovascular diseases except EH.

However, there is no doubt that this study still has many limitations: It will be difficult to merge and analyze the data because there may be many descriptions of the definition of specific outcome indicators in the literature. It reduces the reliability of the sample size and the results; the reliability of the results may be affected because the quality of the literature is low, the sample size is small, and the test process is not standard. The research method of this study still has some shortcomings, which reduces the quality and authenticity of the test.

Researchers should pay more attention to the selection of objective outcome indicators and not excessive use of subjective outcome indicators in the future research; more attention should be paid to the design of the research scheme and the rigor of the trial link in follow-up studies. Clinical trials with rigorous design, standardized process, multi-center and large samples are used to verify the results and provide higher quality evidence.

## Author contributions

**Conceptualization:** Jisen Zhao, Dong Guo.

**Data curation:** Jisen Zhao, Dong Guo, Maoxia Fan, Yongcheng Liu.

**Methodology:** Maoxia Fan.

**Writing – original draft:** Jisen Zhao, Dong Guo, Maoxia Fan, Yongcheng Liu.

**Writing – review & editing:** Jisen Zhao, Dong Guo, Maoxia Fan.
